# The Archaellum of Methanospirillum hungatei Is Electrically Conductive

**DOI:** 10.1128/mBio.00579-19

**Published:** 2019-04-16

**Authors:** David J. F. Walker, Eric Martz, Dawn E. Holmes, Zimu Zhou, Stephen S. Nonnenmann, Derek R. Lovley

**Affiliations:** a Department of Microbiology, University of Massachusetts—Amherst, Amherst, Massachusetts, USA; b Institute for Applied Life Sciences, University of Massachusetts—Amherst, Amherst, Massachusetts, USA; c Department of Physical and Biological Science, Western New England University, Springfield, Massachusetts, USA; d Department of Mechanical and Industrial Engineering, University of Massachusetts—Amherst, Amherst, Massachusetts, USA; University of Delaware; McGill University; University of California, San Franscisco

**Keywords:** protein nanowire, conductive pili, electromicrobiology

## Abstract

Microbially produced electrically conductive protein filaments are of interest because they can function as conduits for long-range biological electron transfer. They also show promise as sustainably produced electronic materials. Until now, microbially produced conductive protein filaments have been reported only for bacteria. We report here that the archaellum of Methanospirillum hungatei is electrically conductive. This is the first demonstration that electrically conductive protein filaments have evolved in *Archaea*. Furthermore, the structure of the M. hungatei archaellum was previously determined (N. Poweleit, P. Ge, H. N. Nguyen, R. R. O. Loo, et al., Nat Microbiol 2:16222, 2016, https://doi.org/10.1038/nmicrobiol.2016.222). Thus, the archaellum of *M. hungatei* is the first microbially produced electrically conductive protein filament for which a structure is known. We analyzed the previously published structure and identified a core of tightly packed phenylalanines that is one likely route for electron conductance. The availability of the *M. hungatei* archaellum structure is expected to substantially advance mechanistic evaluation of long-range electron transport in microbially produced electrically conductive filaments and to aid in the design of “green” electronic materials that can be microbially produced with renewable feedstocks.

## Abstract (precis)

Microbially produced electrically conductive protein filaments are a revolutionary, sustainably produced, electronic material with broad potential applications. The design of new protein nanowires based on the known *M. hungatei* archaellum structure could be a major advance over the current empirical design of synthetic protein nanowires from electrically conductive bacterial pili. An understanding of the diversity of outer-surface protein structures capable of electron transfer is important for developing models for microbial electrical communication with other cells and minerals in natural anaerobic environments. Extracellular electron exchange is also essential in engineered environments such as bioelectrochemical devices and anaerobic digesters converting wastes to methane. The finding that the archaellum of *M. hungatei* is electrically conductive suggests that some archaea might be able to make long-range electrical connections with their external environment.

## OBSERVATION

Electrically conductive pili (e-pili) expressed by microbes in the domain *Bacteria* play an important role in extracellular electron exchange between cells and their extracellular environment (1, 2). e-Pili are found in diverse bacteria (1, 3, 4) but have been studied most extensively in Geobacter sulfurreducens and related *Geobacter* species in which e-pili are essential for long-range electron transport to Fe(III) oxide minerals, interspecies electron transfer, and electron conduction through biofilms (1). e-Pili enable unprecedented long-range (micrometer) electron conduction along the length of a protein filament, which not only has important biological implications but also suggests diverse applications for these “protein nanowires” as a sustainably produced electronic material (1, 5–7). There is substantial debate over the potential mechanisms of long-range electron transport in e-pili (1, 6, 7). Although it has been possible to determine the structure of some pili with cryo-electron microscopy (cryo-EM) (8), an experimentally determined structure of G. sulfurreducens e-pili that could help clarify electron transport mechanisms is not available. However, from the known importance of aromatic amino acids for the conductivity of e-pili (1), synthetic electrically conductive protein nanowires have been designed that are either microbially produced (9) or assembled *in vitro* (10).

The finding that e-pili have independently evolved multiple times in *Bacteria* (3) raised the question of whether conductive protein filaments have ever evolved in *Archaea*. Diverse *Archaea* exchange electrons with their extracellular environment, reducing extracellular electron acceptors or engaging in direct interspecies electron transfer (DIET) with bacteria (2, 11). The alpha-helix filament structure of archaella, as well as the mechanisms for assembly and export, resembles that of type IV pili (8, 12, 13). However, detailed analysis of the Methanospirillum hungatei archaellum also revealed important differences from previously described structures of bacterial pili, such as a lack of an inner channel and a distinct tertiary structure and subunit packing arrangement (13).

### The Methanospirillum hungatei archaellum is electrically conductive.

We chose the methanogen Methanospirillum hungatei for the initial search for an electrically conductive archaellum (e-archaellum) because M. hungatei is capable of reducing extracellular electron acceptors (14), archaellum expression is readily induced in *M. hungatei* (15), and a cryo-EM (3.4-Å) structure of the archaellum is available (13).

Initial screening of the relative conductivity of diverse bacterial pili is typically determined with conductive atomic force microscopy in which samples are deposited on a conductive surface and a conductive tip serves as a translatable top electrode (16–19). Therefore, 100 µl of a culture of *M. hungatei* grown in low-phosphate medium to induce archaellum expression (15) was drop-cast onto highly oriented pyrolytic graphite (HOPG), washed, dried, and then equilibrated at 40% relative humidity for conductivity measurements. This process was designed to mimic physiologically relevant conditions by avoiding chemical alteration of the archaellum structure and determining conductivity of hydrated archaella.

**FIG 1:**
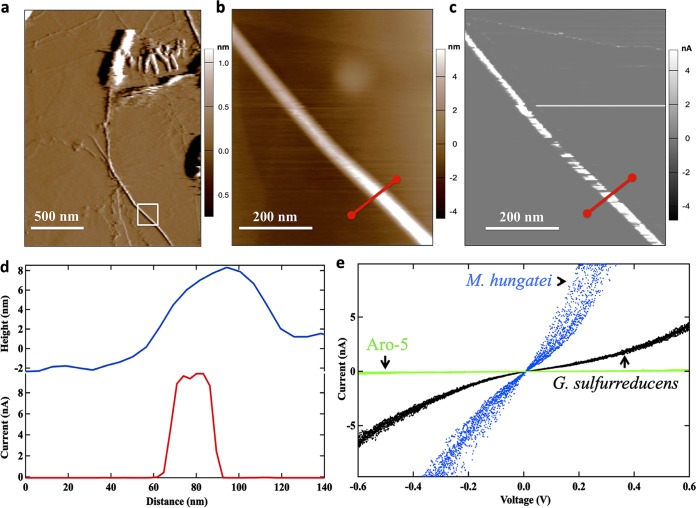
Electrical conductivity of the Methanospirillum hungatei archaellum determined with atomic force microscopy. (a) Contact topographic imaging of *M. hungatei* showing the polar archaellum protruding from the cell. The white box designates the region chosen for additional analysis. (b) Higher-resolution topographic image of the archaellum from the region shown in a white box in panel a. The red line indicates the position for the topographic height and current cross-sectional line profile analysis. (c) Local current image of the individual archaellum with an applied bias of 300 mV. (d) Topographic height and current response from the cross-section designated in panel b. (e) Point-mode current response (I-V) spectroscopy of the individual archaellum (blue). The applied force was 1 nN (see Fig. S3 in the supplemental material). Similar I-V analyses of the wild-type e-pili of G. sulfurreducens (black) and the poorly conductive pili of G. sulfurreducens strain Aro-5 (green) are shown for comparison. A HOPG control is shown in Fig. S4. The *M. hungatei* archaellum conductivity measurement shown is representative of three independent measurements on three archaella (see Fig. S1 and S2 for additional examples).

Cells with a polar archaellum with the expected height of 10 nm (13) were readily detected with topographic imaging in contact mode (Fig. 1a, b, and d). Conductive imaging demonstrated that the archaellum was electrically conductive (Fig. 1c to e; see also Fig. S1 and S2 in the supplemental material). Point-mode current-voltage (I-V) spectroscopy revealed a linear-like response with currents that were higher than at the same voltage with G. sulfurreducens e-pili prepared in the same manner (Fig. 1e). The pili of G. sulfurreducens strain Aro-5, which produces pili specifically designed for low conductivity (20, 21), exhibited very low currents at the same voltages (Fig. 1e). Conductance estimated from the linear portion of the I-V curves yielded conductance estimates of 16.9 ± 3.9 nS (mean ± standard deviation; *n* = 9; three independent points on three separate archaella; 8,000 points of measurement taken for each experimental I-V curve comprised of quadruplicate 0.6-V-bias sweeps) for the archaella, 4.5 ± 0.3 nS for the wild-type G. sulfurreducens pili, and only 0.004 ± 0.002 nS for the Aro-5 pili. The estimated conductance of the wild-type G. sulfurreducens pili was similar to the values found in previous studies that employed a comparable measurement technique (16).

10.1128/mBio.00579-19.1 FIG S1 Electrical conductivity of a replicate Methanospirillum hungatei archaellum determined with atomic force microscopy. (a) Additional example of topological analysis of another representative archaellum. The red line designates the slice taken for the topological height and current cross-sectional line profile analysis in panel c. (b) Current scan with an applied bias of +300 mV to identify a local current of the individual archaellum. (c) Topological height and current response from cross-sectional section designated by a red line in panels a and b. Download FIG S1, PDF file, 1.3 MB. Copyright © 2019 Walker et al. 2019 Walker et al. This content is distributed under the terms of the Creative Commons Attribution 4.0 International license.

10.1128/mBio.00579-19.2 FIG S2 Conductance values from the point-mode current response (I-V) spectroscopy measurements of three Methanospirillum hungatei archaellums. (a) Additional point-mode current response (I-V) spectroscopy measurements of the archaellum identified in Fig. 1. (b) Point-mode current response spectroscopy of the archaellum identified in Fig. S1. (c) Point-mode current response (I-V) spectroscopy of a third archaellum. Calculations were made using a linear fit model between −0.2 V and 0.2 V, and conduction was reported in siemens (S). Download FIG S2, PDF file, 0.5 MB. Copyright © 2019 Walker et al. 2019 Walker et al. This content is distributed under the terms of the Creative Commons Attribution 4.0 International license.

10.1128/mBio.00579-19.3 FIG S3 Current, force, and deflection voltage data recorded during the current response (I-V) spectroscopy measurements. The top panel shows the current response of the I-V curve shown in Fig. 1d. The middle panel shows the continuous force of the AFM tip applied to the top of the archaellum (1 nN). The bottom panel shows the set point (0.02 V) applied to the top of the filament during the I-V curve. Download FIG S3, PDF file, 0.8 MB. Copyright © 2019 Walker et al. 2019 Walker et al. This content is distributed under the terms of the Creative Commons Attribution 4.0 International license.

10.1128/mBio.00579-19.4 FIG S4 Control: point-mode current response (I-V) spectroscopy measurement of the graphite substrate (HOPG). The top panel shows the current response of the I-V curve of HOPG. The middle panel shows the continuous force of the AFM tip applied to the HOPG (1 nN). The bottom panel shows the set point (0.02 V) applied to the top of the HOPG during the I-V curve. Download FIG S4, PDF file, 0.8 MB. Copyright © 2019 Walker et al. 2019 Walker et al. This content is distributed under the terms of the Creative Commons Attribution 4.0 International license.

These results demonstrated that the *M. hungatei* archaellum is conductive and suggest that a search for electrically conductive protein filaments in other *Archaea* as well as the *Eukarya* is warranted. It has been proposed that electrically conductive filaments of anaerobic methane-oxidizing archaea may be conduits for extracellular transfer to electron-accepting partners (22). Other possible benefits of archaellum conductivity might include facilitating attachment by dissipating charge barriers between cells and surfaces or electrical signaling between cells. Expression of synthetic, poorly conductive pili has played an important role in elucidating the function of e-pili in *Geobacter* species (1). Similar functional studies of *M. hungatei* will require the development of genetic tools for this microbe.

### The *M. hungatei* archaellum contains a core of closely packed phenylalanines.

**FIG 2:**
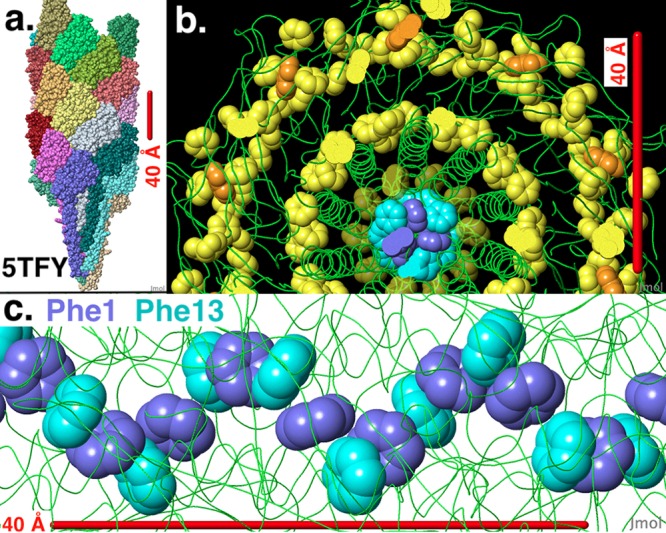
A core chain of tightly packed aromatic rings is evident in the distribution of aromatic amino acids in the structure of the *M. hungatei* archaellum determined previously by Poweleit et al. (PDB accession no. or code 5TFY and EMDB code 8405 [13]). (a) The atomic model 5TFY is an assembly of 26 archaellin protein chains (all atoms shown space filling at van der Waals radii, each chain a distinct color, axis vertical). The cryo-EM map (EMDB code 8405), not shown, spans a larger number of chains, and a complete archaellum consists of ∼61,500 archaellin chains (13). (b) In cross section (axis perpendicular to the image), aromatic rings form three well-separated groups: a core (Phe1 blue, Phe13 cyan, Phe20 dim yellow), a middle sleeve, and an outer sleeve (Phe and Tyr yellow; Trp orange). (c) Tightly packed core of alternating Phe1 (blue) and Phe13 (cyan) rings (axis horizontal). Ring center distances are 4.5 and 5.1 Å. Phe20, shown in dim yellow in panel b, is not shown in panel c due to wider spacing and positioning peripheral to the core chain of Phe1 and Phe13. Protein main chain traces are shown in green in panels b and c. Images and measurements were made with Jmol.Org.

The cryo-EM structure of the *M. hungatei* e-archaellum (Fig. 2a), previously reported by Poweleit et al. (13), provides a much needed first opportunity to directly evaluate possible routes for long-range electron transport along a biologically produced protein filament. Aromatic rings of phenylalanine, tyrosine, and tryptophan are grouped into three well-separated regions: an outer sleeve (Fig. 2b and Fig. S5a), a middle sleeve (Fig. 2b and Fig. S5b), and a core (Fig. 2c). It was previously noted that the N-terminal phenylalanine residues in the archaellin subunits (Phe1) interact to “create a spokes effect via a π-stacking sandwich” that plays a key role in stabilizing the structure (13). Additional analysis of the distribution of aromatic amino acids (Fig. 2b and c and Fig. S5) further revealed that the aromatic rings of Phe1 and Phe13 in the core of the structure are packed almost as close as is physically possible (distances between ring centers of Phe1 and Phe13 of 4.5 and 5.1 Å), with angled T-shaped geometric orientations, which previous studies have suggested may enable π-π interactions (23). Furthermore, recent experimental evidence has indicated that, even in the absence of π-π stacking, phenylalanines within the hydrophobic core of an amino acid α-helical structure can facilitate long-range electron transport (10, 24). Therefore, our working hypothesis is that the Phe1-Phe13 core is at least one of the features contributing to the e-archaellum conductivity. Other aromatic amino acids of note include Phe20 (Fig. 2b), which is positioned close to the Phe1-Phe13 core, as well as outer and middle sleeves of aromatics that are well separated from each other and from the Phe1,13,20 core (Fig. 2b). Unlike the core, the outer and middle aromatic sleeves lack any closely spaced continuous chain of aromatics extending the length of the filament (Fig. S5).

10.1128/mBio.00579-19.5 FIG S5 Absence of any continuous chain of aromatic rings in the outer and middle sleeves of aromatic amino acids in the structure of the *M*. *hungatei* archellum determined previously by Poweleit et al. (PDB code 5TFY and EMDB code 8405 [13]). (a) Side view (axis horizontal) of the outer sleeve of aromatic rings (rear half of model hidden; Phe and Tyr yellow; Trp orange). (b) Side view (axis horizontal) of the middle sleeve of aromatic rings (rear half of model hidden; Phe and Tyr yellow). Protein main chain traces are shown in green. The images were made with Jmol.Org. Download FIG S5, PDF file, 0.7 MB. Copyright © 2019 Walker et al. 2019 Walker et al. This content is distributed under the terms of the Creative Commons Attribution 4.0 International license.

Analogous to recent studies of G. sulfurreducens e-pili (20, 21, 25, 26), genetic manipulations to alter the positions of aromatic amino acids or other amino acids that may promote conductivity within the *M. hungatei* archaellum could lead to a better understanding of the structural features contributing to conductivity. The added benefit of such studies with the *M. hungatei* e-archaellum is that it will be possible to directly examine structural modifications to electron conductance pathways with cryo-EM. In the absence of genetic tools for *M. hungatei*, it will be necessary to heterologously express the gene for the *M. hungatei* archaellin in a genetically tractable archaeal host, similar to the expression of heterologous e-pili in G. sulfurreducens (3) or to identify a similar e-archaellum in a genetically tractable archaeon.

Microbially produced protein nanowires show substantial promise as a sustainable “green” electronic material with possibilities for functionalization and biocompatibility not available with other nanowire materials (1, 5–7). e-Archaella offer a unique opportunity to directly examine how synthetic designs to tune conductivity and/or add functionality influences protein nanowire structure, enabling a less empirical approach to the design of protein nanowire electronics.

### Methods.

*M. hungatei* was grown as previously described (1) in low-phosphate medium to induce archaellum expression. An aliquot (100 μl) of the culture was drop-cast onto highly oriented pyrolytic graphite (HOPG). Cells were allowed to attach to the HOPG for 10 min, and then the liquid was removed with a pipette tip. The surface was washed twice with 100 μl of deionized water, the surface was blotted dry at the edge with a Kimwipe, and the sample was placed in a desiccator overnight. All samples were equilibrated with atmospheric humidity (40%) inside the atomic force microscope (AFM) chamber for at least 2 h at 26.1°C at 1.1 mbarg. Conductive atomic force microscopy was performed using an Oxford Instruments/Asylum Research Cypher ES atomic force microscope. All topographic and current imaging was performed with a Pt/Ir-coated Arrow-ContPT tip with a 0.36701-N/m spring constant (NanoWorld AG, Neuchâtel, Switzerland). Topographic imaging was performed at a force of 0.1 nN. The conductive tip was attached to an ORCA dual-gain transimpedance amplifier and held at ground to serve as a translatable top electrode. A 300-mV bias was applied to the HOPG, and the locally detected current response of the archaellum was identified. Point-mode current-voltage (I-V) spectroscopy was performed by applying the conducting AFM tip at a force of 1 nN to the top of the archaellum and performing a voltage sweep at a frequency of 0.99 Hz. Continual force and current responses were collected for each I-V curve (Fig. S3) to ensure good consistent contact with the sample and avoid archaellum damage. The HOPG was periodically touched between samples to ensure the correct I-V response and tip quality (Fig. S4). Conductance was calculated from the linear portion of each I-V curve (−0.2 V to 0.2 V) as previously described (2). Average conductance and standard deviation were calculated for each of the three independent points on the three independent archaella.
