# Supplementary figures and images for: The Archaellum of Methanospirillum hungatei Is Electrically Conductive
Source: mBio. 2019 Apr 16;10(2):e00579-19. doi: 10.1128/mBio.00579-19 (PMC6469973; doi:10.1128/mBio.00579-19)

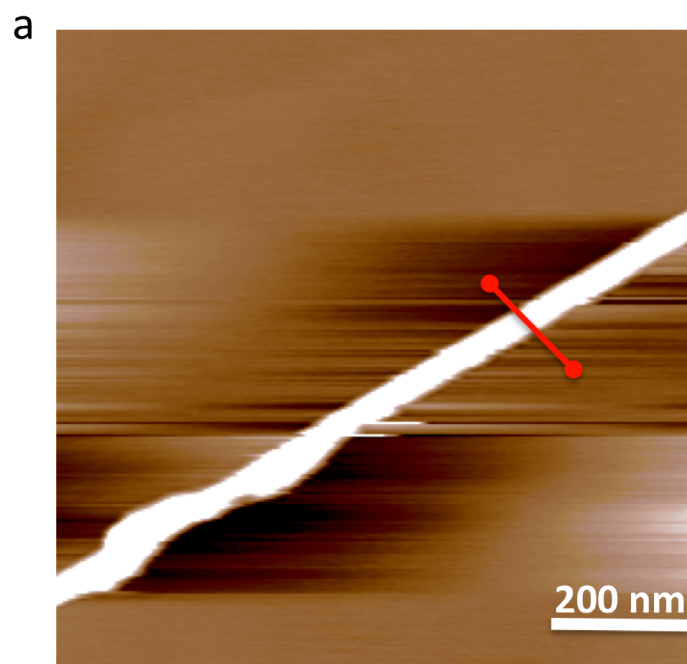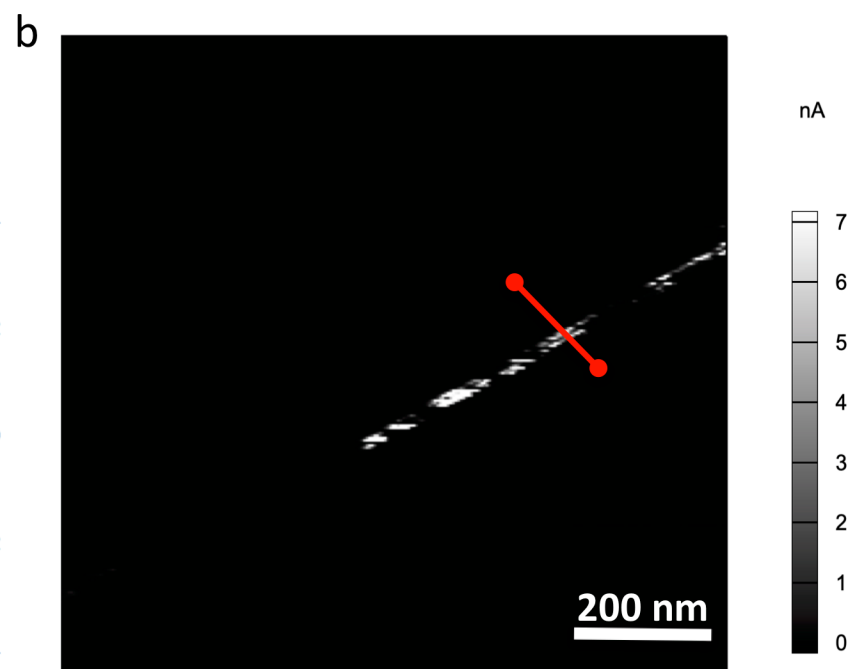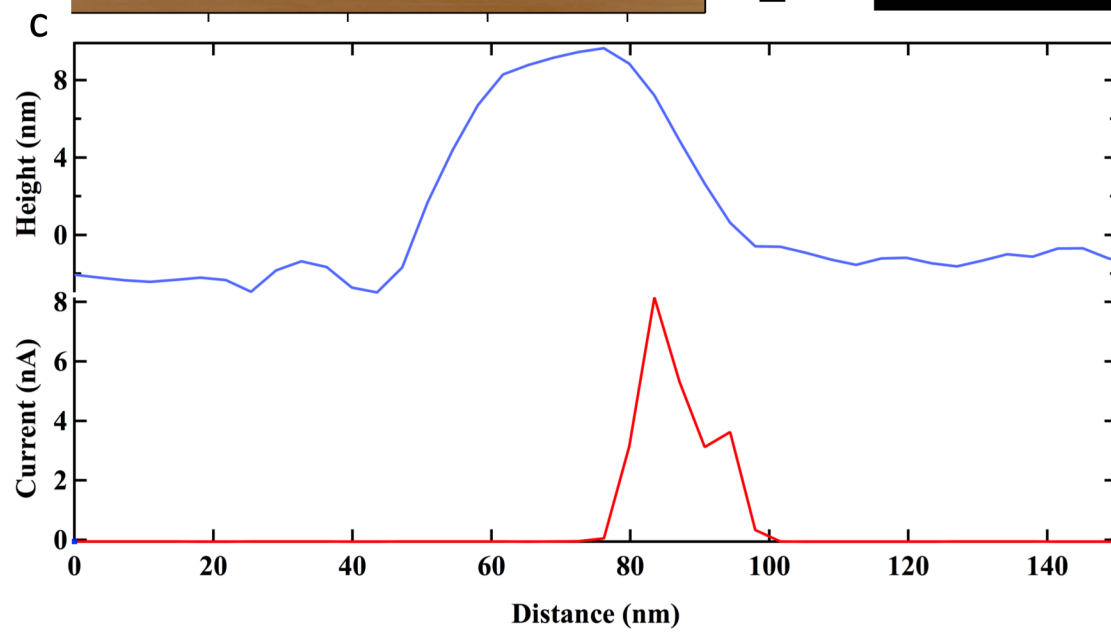

Supplement: FIG S1 [file mBio.00579-19-sf001.pdf]

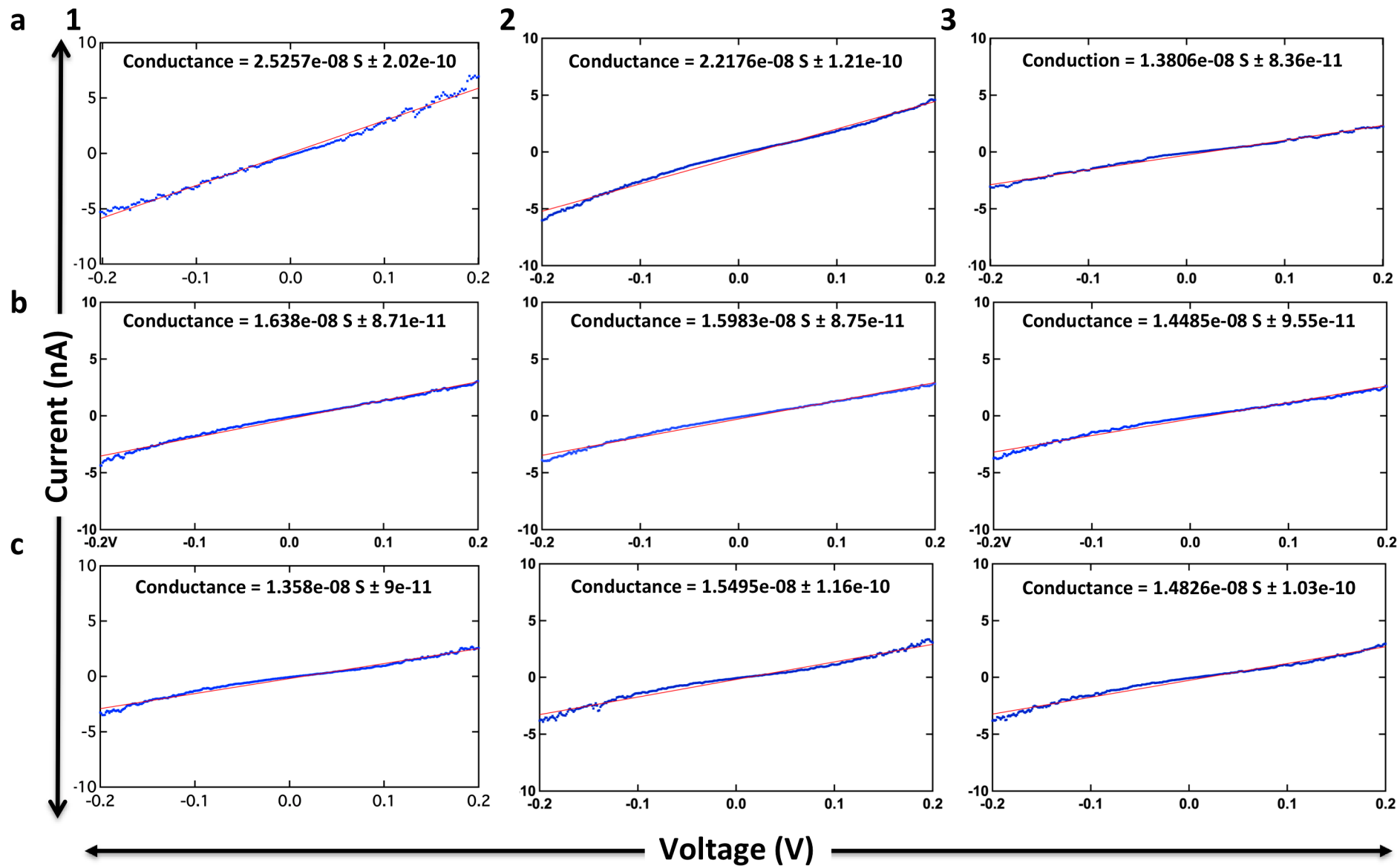

Supplement: FIG S2 [file mBio.00579-19-sf002.pdf]

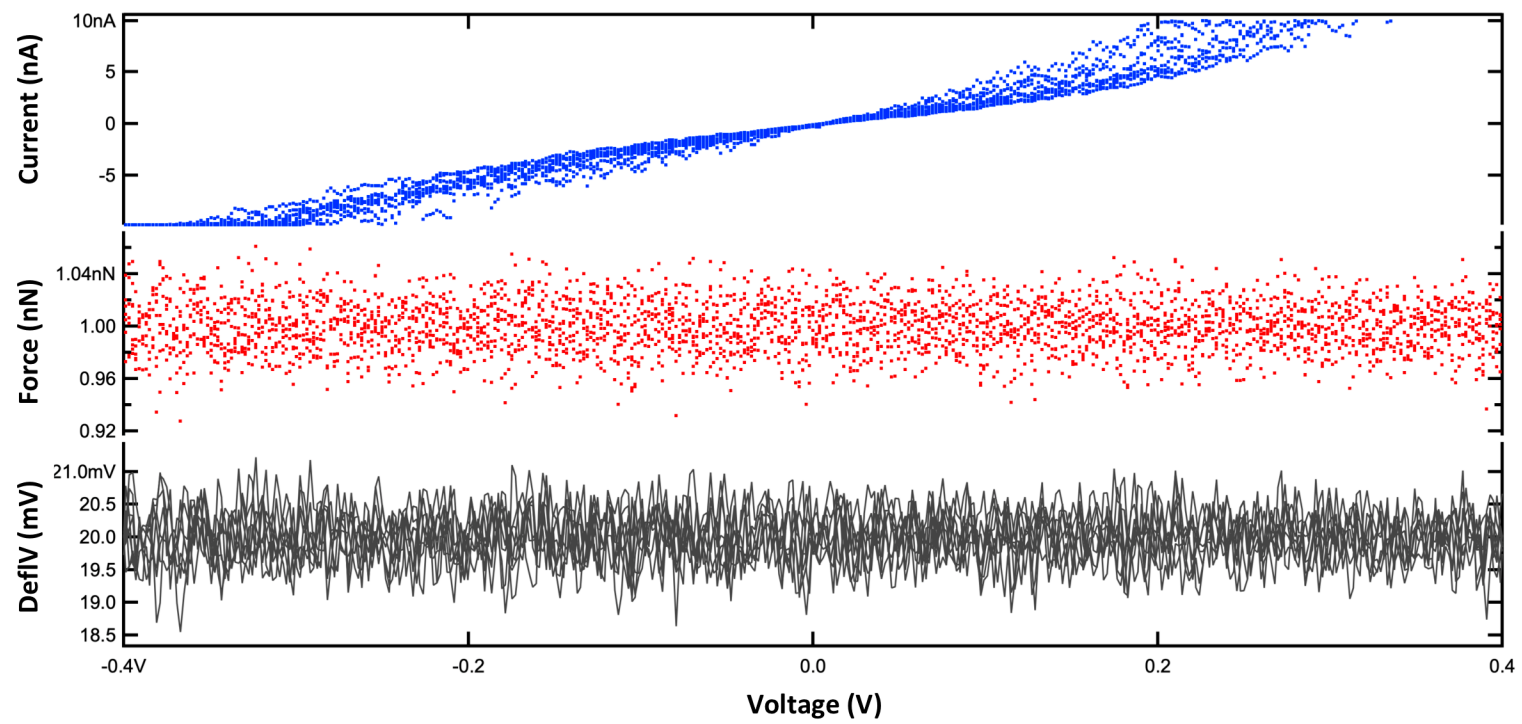

Supplement: FIG S3 [file mBio.00579-19-sf003.pdf]

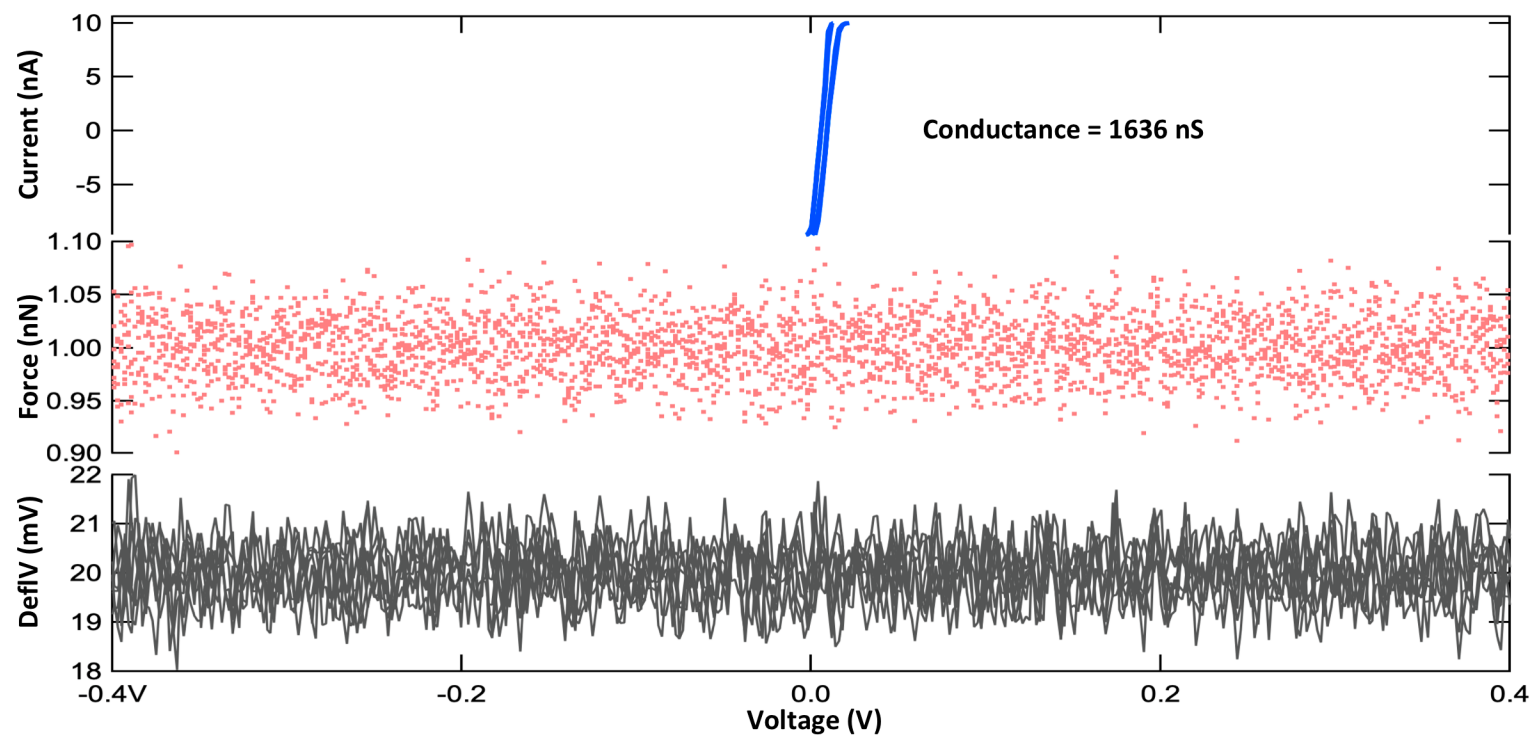

Supplement: FIG S4 [file mBio.00579-19-sf004.pdf]

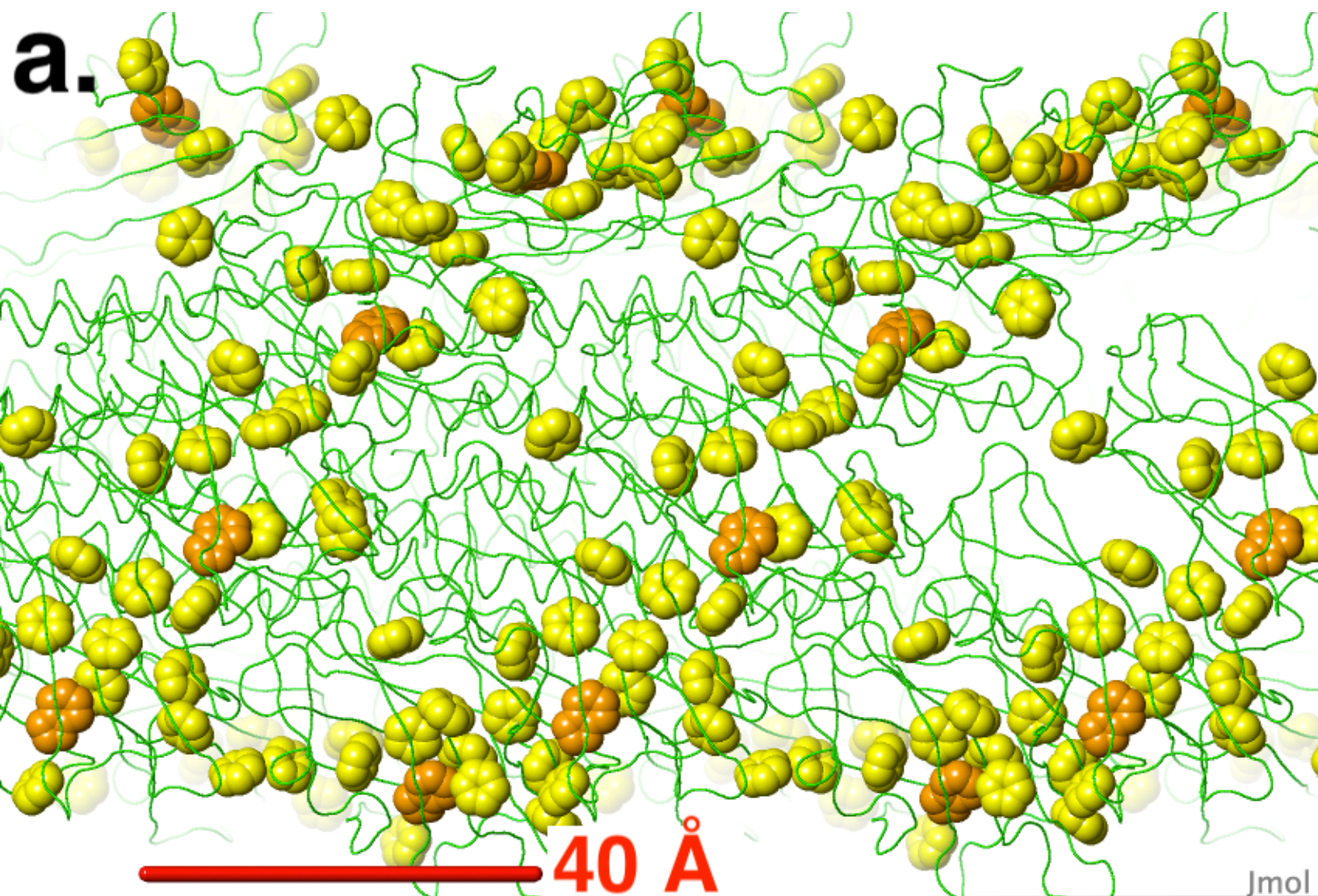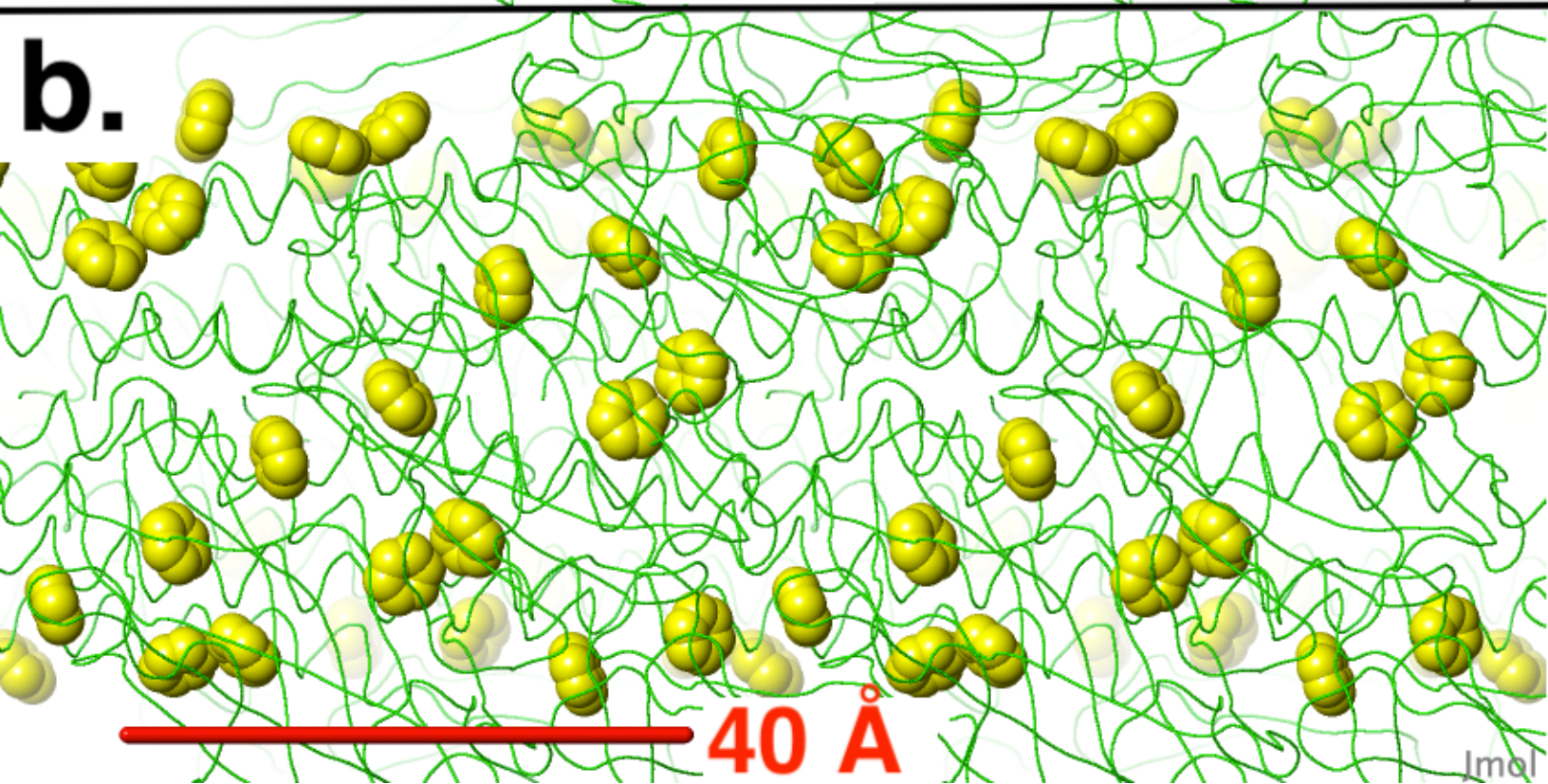

Supplement: FIG S5 [file mBio.00579-19-sf005.pdf]
